# Systolic Blood Pressure Trajectories and the Progression of Arterial Stiffness in Chinese Adults

**DOI:** 10.3390/ijerph191610046

**Published:** 2022-08-15

**Authors:** Run Liu, Dankang Li, Yingping Yang, Yonghua Hu, Shouling Wu, Yaohua Tian

**Affiliations:** 1 Ministry of Education Key Laboratory of Environment and Health and State Key Laboratory of Environmental Health (Incubating), School of Public Health, Tongji Medical College, Huazhong University of Science and Technology, No. 13 Hangkong Road, Wuhan 430030, China; 2 Department of Maternal and Child Health, School of Public Health, Tongji Medical College, Huazhong University of Science and Technology, No. 13 Hangkong Road, Wuhan 430030, China; 3 Department of Epidemiology and Biostatistics, School of Public Health, Peking University, No. 38 Xueyuan Road, Beijing 100191, China; 4 Department of Cardiology, Kailuan Hospital, North China University of Science and Technology, No. 57 Xinhua East Road, Tangshan 063001, China

**Keywords:** SBP, trajectories, baPWV, arterial stiffness, longitudinal study

## Abstract

Evidence on the association between systolic blood pressure (SBP) trajectories and arterial stiffness progression is scarce. The current study aimed to identify the association between SBP trajectories and the progression of arterial stiffness over time in Chinese adults. This study included 30,384 adult participants. Latent mixture modeling was used to identify the SBP trajectory patterns from 2006 to 2010. The brachial–ankle pulse wave velocity (baPWV) was used to measure arterial stiffness. The associations between SBP trajectories and the progression of arterial stiffness were explored using multiple linear regression models. We identified five distinct SBP trajectories and took the low-stable group as the reference. In the cross-sectional analysis, the four SBP trajectories were significantly associated with higher baPWV levels (*p* < 0.001) compared with the reference. In the longitudinal analysis, after adjusting for covariates and the baseline baPWV, the SBP trajectories were significantly associated with the progression of the baPWV, with corresponding β (95% CI) values of 23.3 (17.2–29.5) cm/s per year for the moderate-stable group, 44.8 (36.6–52.9) cm/s per year for the moderate-increasing group, 54.6 (42.2–67.0) cm/s per year for the elevated-decreasing group, and 66.8 (54.7–79.0) cm/s per year for the elevated-stable group. Similar significant results were also observed in the non-hypertensive population. In conclusion, SBP trajectories were related to the baseline baPWV and the progression of the baPWV. Blood pressure control may be considered a therapeutic target to further reduce the risk of arterial stiffness.

## 1. Introduction

Arterial stiffness, which is an early detectable manifestation of adverse structural and functional changes in the vessel wall, can directly damage the cardiovascular system by lowering arterial elasticity [1]. Increased arterial stiffness is considered to be an independent risk factor for cardiovascular disease (CVD) [2,3,4]. Brachial–ankle pulse wave velocity (baPWV) is a noninvasive technique that is used as an indicator in assessing the degree of central and peripheral arterial stiffness and has been widely applied for the assessment of vascular risk in the general population because of its simplicity and high reproducibility [5,6].

Blood pressure (BP) is one of the most powerful risk factors for arterial stiffness and provides a strong predictive value for the future progression of arterial stiffness. Growing evidence has found that poor BP control was strongly associated with an increased risk of arterial stiffness [7,8]. For example, Finnish research demonstrated that adults with a high SBP had an increased risk of high PWV [9]. In addition, BP indices were identified to be independently and positively associated with the baPWV and elevated baPWV in treated hypertensive patients [8]. Another study in Chinese hypertensive adults also indicated that higher SBP would lead to a higher risk of progressive arterial stiffness [10]. However, most reported studies about the topic were on the basis of a single measurement of SBP but did not consider the effect of the change in SBP over time. In fact, SBP levels change over time. Neglecting the time-varying and cumulative mean value of SBP over time would result in regressing to the null hypothesis of the real association between SBP and arterial stiffness risk. Recently, increasing attention has been paid to the effect of change in SBP. Longitudinal cohort studies showed that blood pressure control was associated with arterial stiffness progression in hypertensive individuals [11,12,13]. Several studies used an assessment of trajectories based on multiple valid measurements to reflect long-term BP patterns and demonstrated the effects of SBP trajectories on cardiovascular outcomes [14,15]. However, no study to date has explored the association between SBP trajectories and the progression of arterial stiffness.

Therefore, we used data from a community-based cohort study with several SBP measures over several years to explore cross-sectional and longitudinal associations between SBP trajectories and the baseline baPWV and its progression.

## 2. Methods

### 2.1. Study Design and Participants

The Kailuan Study launched in Tangshan City, China, is an ongoing prospective cohort study. The study design and procedures were described elsewhere [16,17]. Briefly, from June 2006 to October 2007, a total of 101,510 participants aged ≥18 years at the Kailuan Company agreed to participate and were enrolled. All participants completed questionnaire interviews, laboratory tests, and clinical examinations at the time of enrollment and have been followed up every two years since 2006 [16,17].

In the present analysis, we set the study baseline at the first baPWV examination. Participants were included if they participated in at least one survey from 2010 to 2017 and agreed to undergo a baPWV examination at the same time. Among the 31,963 participants who met the criteria, we excluded participants with a history of CVD or cancer at baseline (*n* = 1247), and participants with lacking data on SBP between 2006 and 2010 and their baseline baPWV (*n* = 332). Finally, a total of 30,384 participants were included (Supplementary Figure S1). The Ethics Committees of Kailuan General Hospital provided the study protocol and written informed consent was obtained from all the participants.

### 2.2. Measurement of Blood Pressure

In 2006, 2008, and 2010, trained physicians and nurses conducted face-to-face examinations [18]. A mercury sphygmomanometer with an appropriately sized cuff was used to measure blood pressure (BP) following standard steps [19]. The appearance of the first two or more Korotkoff sounds was used to define systolic blood pressure (SBP) and diastolic blood pressure (DBP) was defined as the point where the Korotkoff sound disappeared. After resting for at least 5 min for each participant, the BP was measured 3 times, and the average value was used as the final measurement.

### 2.3. Measurement of baPWV

A BP-203 RPE III networked arterial stiffness detection device was used to measure the baseline and progressive baPWV. The detailed procedures were reported [5,20]. In brief, the lower edge of the arm sleeve was 2–3 cm above the transverse line of the elbow fossa, and the lower edge of the ankle sleeve was 1–2 cm above the upper part of the medial malleolus. A phonocardiogram was placed on the left edge of the sternum, and electrocardiogram electrodes were placed on both wrists. To minimize the effects of sympathetic activity on the baPWV measurements, participants lay in a supine position (in a room maintained at 22–25 °C) for at least 5 min prior to the measurement. Measurements of the baPWV were taken twice and the mean value was used as the final measurement. The maximum of the right and left baPWV was used for the present analysis. The progression of arterial stiffness was defined as follows: the baPWV value at follow-up minus the value at baseline, and then divided by the duration of the follow-up (years).

### 2.4. Assessment of Covariates

Demographic data (e.g., age, sex, education) and lifestyle behaviors (e.g., alcohol drinking and smoking) were collected using self-administered questionnaires during interviews. On the basis of previous scientific literature [14,15,21], the following variables were included as covariates: age, sex (female or male), educational level (low, intermediate, or high), physical activity (inactive or active), smoking status (never, former, or current smokers), alcohol drinking status (never, former, or current alcohol drinkers). The body mass index (BMI) was calculated as weight in kilograms divided by height in meters squared. After fasting for at least 10 h, the venous blood samples of participants were collected in the morning. The serum total cholesterol (TC) and fasting blood glucose (FBG) were measured. The mean arterial BP (MAP) was calculated as 1/3 × SBP + 2/3 × DBP.

### 2.5. Statistical Analysis

In the current study, SBP trajectories were used as exposure. We used latent mixture modeling to identify subgroups that shared similar potential SBP trajectories from 2006 to 2010 [22,23]. The Bayesian information criterion (BIC) was used to evaluate the model fit, and the smallest negative number indicated the best-fitting model. By comparing the BICs of models with 1, 2, 3, 4, and 5 trajectories, we found that the best fit model was the model with 5 trajectories. We then compared models with different functional forms. Starting with the highest polynomial, we considered and evaluated the cubic, quadratic, and linear terms on the basis of their significance level (*p* < 0.05). Finally, the model with 5 trajectories identified the fit best.

We identified five distinct SBP trajectories and took the low-stable group as the reference group. Multivariate linear regression models were used to explore the associations. In the cross-sectional analysis, covariates for adjustment were selected a priori, including age, gender, education level, smoking status, alcohol drinking status, physical activity, BMI, MAP, FBG, and TC. Then, we further explored the longitudinal associations when adjusting for covariates above and at the baseline baPWV. The missing values for covariates were imputed via multiple imputations using chain equations. We conducted several sensitivity analyses to assess the robustness of our results: we analyzed the association between age and gender groups, and as participants with hypertension may affect the BP trajectory, we conducted analyses by excluding participants diagnosed with hypertension at baseline.

All statistical analyses were conducted using SAS (version 9.4; SAS Institute Inc., Cary, NC, USA). A two-sided *p* < 0.05 was considered statistically significant.

## 3. Results

### 3.1. Characteristics of Participants

The current study enrolled 30,384 participants with BP measurements in the final data analysis (Supplementary Figure S1). It should be noted that there were small, albeit statistically significant, differences for most of the baseline characteristics between participants with and without repeated baPWV measurements (Supplementary Table S1). Demographic and baseline clinical characteristics information for the five trajectory groups is shown in Table 1. Individuals in the low-stable group were used as the reference. Compared with the other four groups, individuals in the low-stable group were the most likely to be younger, female, more highly educated, and exercise more. Meanwhile, the proportions of never alcohol intake and never smoking were higher. The mean values of BMI, MAP, FBG, and TC were also lower in the low-stable group.

### 3.2. Association between SBP Trajectories and baPWV—A Cross-Sectional Analysis

**Figure 1:**
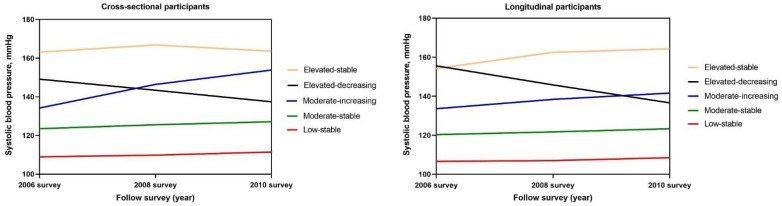
Systolic blood pressure (SBP) trajectory patterns for general participants from 2006 to 2010. SBP was classified into five groups according to the latent mixture modeling. The red line indicates the low-stable pattern, the green line indicates the moderate-stable pattern, the blue line indicates the moderate-increasing pattern, the black line indicates the elevated-decreasing pattern, and the yellow line indicates the elevated-stable pattern. The x-axis refers to the years of follow-up surveys of the participants. The y-axis refers to the SBPs of the participants.

A total of 30,384 participants were included in the cross-sectional analysis. Among these participants, five SBP trajectories over 4 years were identified (Figure 1). Altogether, 2437 (8.02%) participants starting at moderate levels and experiencing a rapid BP increase were placed in the moderate-increasing group; 3727 (12.27%) participants starting at elevated levels and experiencing a rapid BP decrease were placed in the elevated-decreasing group; and participants maintaining low, moderate, or elevated BP during the follow-up period were divided into the low-stable group (7945, 26.15%), moderate-stable group (14,995, 49.35%), and elevated-stable group (1280, 4.21%), respectively.

The associations of SBP trajectories with the baseline baPWV are listed in Table 2. In the multivariate-adjusted model, compared with the reference, the mean levels of the four SBP trajectories were significantly associated with the baPWV levels (*p* < 0.001) and had 109.3 (99.5–119.1) cm/s, 279.4 (263.4–295.3) cm/s, 199.6 (181.1–218.0) cm/s, and 323.7 (298.5–348.9) cm/s higher increases in the baseline baPWV in the moderate-stable, moderate-increasing, elevated-decreasing, and elevated-stable group, respectively.

### 3.3. Association between SBP Trajectories and Progression of baPWV—A Longitudinal Analysis

A total of 14,223 participants with repeated baPWV measurements were included in the longitudinal analysis. We investigated the longitudinal associations of the SBP trajectories with the baPWV progression over a mean follow-up of 3.1 years after adjusting for the baseline baPWV and other risk factors (Table 3). The numbers of individuals in the moderate-increasing group, elevated-decreasing group, low-stable group, moderate-stable group, and elevated-stable group were 2825, 728, 3350, 6631, and 689, respectively.

Compared with the low-stable group, the levels of SBP trajectories were significantly associated with the progression of the baPWV, with corresponding β (95% CI) values of 23.3 (17.2–29.5) cm/s per year for the moderate-stable group, 66.8 (54.7–79.0) cm/s per year for the elevated-stable group, 44.8 (36.6–52.9) cm/s per year for the moderate-increasing group, and 54.6 (42.2–67.0) cm/s per year for the elevated-decreasing group.

### 3.4. Sensitivity Analyses

**Figure 2:**
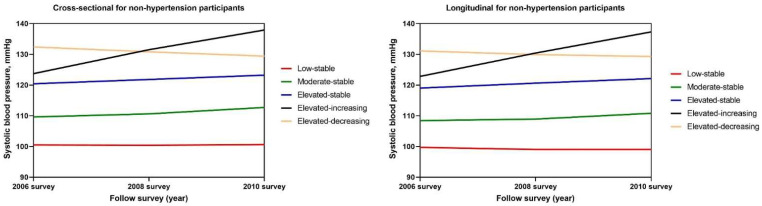
Systolic blood pressure (SBP) trajectory patterns for non-hypertensive participants from 2006 to 2010. SBP was classified into five groups according to the latent mixture modeling. The red line indicates the low-stable pattern, the green line indicates the moderate-stable pattern, the blue line indicates the elevated-stable pattern, the black line indicates the elevated-increasing pattern, and the yellow line indicates the elevated-decreasing pattern. The x-axis refers to the years of follow-up surveys of the participants. The y-axis refers to the SBPs of the participants.

As shown in Table 3 and Figure 2, we identified five SBP trajectories patterns and explored the associations after excluding participants diagnosed with hypertension. Similar to the findings in the general population, we found that with the increasing mean levels of SBP trajectories, the levels of progressive baPWV also increased. Furthermore, the elevated-decreasing group had a lower risk of arterial stiffness progression than the elevated-increasing group, though the mean levels of SBP showed no significant difference (Table 3 and Figure 2). Moreover, positive significant associations were also observed in the age and gender subgroups (Supplementary Tables S2–S5 and Figures S2–S5).

## 4. Discussion

To the best of our knowledge, this is the first study that assessed the effect of the SBP trajectories on the progression of arterial stiffness. In the current study based on a prospective cohort in Kailuan, we observed five different SBP trajectories in a total of 30,384 participants over 4 years of follow-ups. In both the cross-sectional study and longitudinal study, higher levels of SBP trajectories were associated with higher levels of baseline baPWV and progressive baPWV. Additionally, we also modeled the trajectories in non-hypertensive participants and generated similar significant results.

Previous studies were mostly conducted based on a single measurement of SBP, which is not comprehensive. Therefore, considering the effect of changes in long-term BP, recent studies began to assess blood pressure levels in other ways, such as BP trajectories [15,24], the BP variability [25,26], and grouping subjects according to changes in their hypertension status [13]. So far, studies have shown that the long-term BP level is an important determinant of arterial stiffness progression [13,27,28,29]. Studies found that a lower BP and a well-controlled BP increase can slow down a baPWV increase in hypertensive individuals [13], and long-term adequate control of BP can slow arterial stiffening [28]. Another study suggested that the adverse effects of early high SBP on late vascular damage development can be reversed by late control of SBP [29]. Furthermore, BP trajectories were used to reflect the effect of long-term BP patterns on cardiovascular outcomes [14,15]. Based on previous studies, our study used trajectories assessment to reflect long-term SBP changes and explore the relationship of SBP trajectories with the arterial stiffness progression in both cross-sectional and longitudinal analyses. Furthermore, similar positive associations were observed in both analyses. Among the three stable SBP groups, with the increasing mean levels of SBP trajectories, the levels of the baseline baPWV and progressive baPWV increased. Furthermore, in the analysis of non-hypertensive participants, we also observed that although the cumulative mean levels of the trajectories of the elevated-increasing group with a lower baseline SBP and the elevated-decreasing group with a higher baseline SBP showed no significant difference, the baseline and progressive baPWVs of the former group increased more. This suggested that using a single SBP value could misclassify risk groups and SBP trajectories provide more insight into the evolving risk of arterial stiffness progression.

The mechanisms of the effect of the SBP trajectories on the progression of arterial stiffness are still being investigated. Studies reported that the adverse effects of increased SBP may be related to a greater traumatic effect of wider BP swings on the vessel wall, promoting early target-organ damage [27,30]. Moreover, independent of the average SBP, the high SBP levels may promote the formation of arterial stiffness in animal models by causing damage to endothelial function, restraining the production of nitric oxide and enhancing the formation of tunica intima [31]. In addition, subgroup analysis can make better use of data to reveal underlying truths. We found that an interaction existed and significant associations were observed in both subgroups. Yet we cannot draw the same conclusion as previous studies: the effect of SBP trajectories on arterial stiffness progression can be stronger in the older age group because of the different SBP trajectory groups in the age subgroups (Supplementary Tables S2 and S3 and Figures S2 and S3). According to previous studies, the underlying mechanism might be that with the increase in age, long-term exposure to high SBP may lead to damage to endothelial function and changes in blood cell endothelial interaction [32,33], resulting the increased stiffness and decreased compliance of the large elastic artery. Similar results were observed in other studies [34,35].

The strengths of our study include the large sample size, the application of a trajectory method, and the use of longitudinal measure data for baPWV. There were some limitations that need to be acknowledged. First, observational studies cannot evaluate the causality of the findings. Second, the baPWV was used instead of the gold standard of the carotid-femoral pulse wave velocity as a biomarker for arterial stiffness. However, the baPWV and the carotid-femoral pulse wave velocity are strongly correlated with each other and were validated for use in large-scale epidemiological cohorts for simple, reproducible, and noninvasive measurements [36,37]. Third, the longitudinal trajectories of SBP were only assessed over an approximately four-year period with measurements at three time points. A longer period of trajectories and a more precise assessment of change patterns should be considered in further studies. Fourth, although the analyses were adjusted for known potential sources of bias, the possibility of unmeasured confounding and reverse causation remains. Lastly, the participants of the Kailuan cohort are mainly Chinese workers, and the proportion of men was about 80%. However, we found positive results in both the male and female subgroups. Whether the observed associations can be applied to other populations and ethnic groups deserves further exploration.

## 5. Conclusions

SBP trajectories were related to the baseline baPWV and the progression of the baPWV. Similar significant results were also observed in a non-hypertensive population. Blood pressure control may be considered a therapeutic target to further reduce the risk of arterial stiffness.

## Figures and Tables

**Table 1:** Demographic and baseline clinical characteristics according to systolic blood pressure (SBP) trajectories (*n* = 30,384).

Variables	Low-Stable Group	Moderate-Stable Group	Moderate-Increasing Group	Elevated-Decreasing Group	Elevated-Stable Group
No. of participants	7945	14,995	2437	3727	1280
Age, years	43.5 ± 10.0	48.6 ± 10.9	55.3 ± 11.3	54.4 ± 11.4	58.6 ± 11.1
Gender, *n* (%)					
Male	3667 (46.2)	11,532 (76.9)	1956 (80.3)	3001 (80.5)	1044 (81.6)
Female	4278 (53.8)	3463 (23.1)	481 (19.7)	726 (19.5)	236 (18.4)
Education level, *n* (%)					
Low	180 (2.3)	649 (4.3)	218 (9.0)	272 (7.3)	167 (13.1)
Intermediate	4031 (50.7)	9527 (63.6)	1628 (66.7)	2687 (72.1)	874 (68.2)
High	3734 (47.0)	4819 (32.1)	591 (24.3)	768 (20.6)	239 (18.7)
Physical activity, *n* (%)					
Inactive	7234 (91.1)	13,202 (88.0)	2023 (83.0)	3076 (82.5)	1020 (79.7)
Active	711 (8.9)	1793 (12.0)	414 (17.0)	651 (17.5)	260 (20.3)
Smoking, *n* (%)					
Never	5621 (70.8)	8372 (55.8)	1315 (54.0)	2193 (58.8)	675 (52.7)
Former	268 (3.3)	829 (5.4)	198 (8.1)	239 (6.4)	118 (9.2)
Current	2056 (25.9)	5794 (38.6)	924 (37.9)	1295 (34.8)	487 (38.1)
Drinking, *n* (%)					
Never	4999 (62.9)	7468 (49.8)	1195 (49.0)	2032 (54.5)	649 (50.7)
Former	167 (2.2)	482 (3.2)	125 (5.2)	159 (4.3)	58 (4.5)
Current	2779 (34.9)	7045 (47.0)	1117 (45.8)	1536 (41.2)	573 (44.8)
BMI, kg/m^2^	23.2 ± 3.0	25.1 ± 3.2	26.0 ± 3.2	26.4 ± 3.2	26.6 ± 3.3
MAP, mmHg	86.2 ± 5.0	98.7 ± 5.2	110.0 ± 5.7	111.2 ± 6.0	121.6 ± 7.1
FBG, mmol/L	5.0 ± 0.9	5.4 ± 1.3	5.3 ± 1.5	5.7 ± 1.6	5.9 ± 1.8
TC, mmol/L	4.7 ± 0.9	4.9 ± 1.0	5.1 ± 1.0	5.1 ± 1.1	5.3 ± 1.0

Abbreviations: BMI, body mass index; MAP, mean arterial pressure; FBG, fasting blood glucose; SBP, systolic blood pressure; TC, total cholesterol. Continuous variables are displayed as mean ± standard deviation (SD), and categorical variables are displayed as number (percentage).

**Table 2:** Associations of SBP trajectories with the baseline baPWV and progressive baPWV.

Trajectory Patterns	No. of Participants	Model 1		Model 2	
		β (95% CI)	*p*-Value	β (95% CI)	*p*-Value
Baseline baPWV (*n* = 30,384)					
Low-stable group	7945	Ref.		Ref.	
Moderate-stable group	14,995	229.4 (220.9–237.9)	<0.001	109.3 (99.5–119.1)	<0.001
Moderate-increasing group	2437	510.1 (495.9–524.3)	<0.001	279.4 (263.4–295.3)	<0.001
Elevated-decreasing group	3727	455.8 (443.7–468.0)	<0.001	199.6 (181.1–218.0)	<0.001
Elevated-stable group	1280	663.9 (645.4–682.3)	<0.001	323.7 (298.5–348.9)	<0.001
Progression of AS (*n* = 14,223)					
Low-stable group	3350	Ref.		Ref.	
Moderate-stable group	6631	31.9 (26.2–37.5)	<0.001	23.3 (17.2–29.5)	<0.001
Moderate-increasing group	2825	61.4 (54.0–68.8)	<0.001	44.8 (36.6–52.9)	<0.001
Elevated-decreasing group	728	72.4 (60.5–84.2)	<0.001	54.6 (42.2–67.0)	<0.001
Elevated-stable group	689	84.8 (73.3–96.3)	<0.001	66.8 (54.7–79.0)	<0.001

Abbreviations: β, regression coefficient; baPWV, brachial–ankle pulse wave velocity; SBP, systolic blood pressure. Model 1 for the baseline baPWV was unadjusted. Model 2 for the baseline baPWV was adjusted for age, gender, education level, smoking status, alcohol drinking status, physical activity, BMI, MAP, FBG, and TC. Model 1 for the progressive baPWV was adjusted for the baseline baPWV. Model 2 for the progressive baPWV was adjusted for age, gender, education level, smoking status, alcohol drinking status, physical activity, baseline baPWV, BMI, MAP, FBG, and TC.

**Table 3:** Associations of the SBP trajectories with the baseline baPWV and progressive baPWV in non-hypertensive participants.

Trajectory Patterns	No. of Participants	Model 1		Model 2	
		β (95% CI)	*p*-Value	β (95% CI)	*p*-Value
Baseline baPWV (*n* = 20,675)					
Low-stable group	907	Ref.		Ref.	
Moderate-stable group	6465	144.7 (125.8–163.5)	<0.001	72.7 (55.3–90.2)	<0.001
Elevated-stable group	11,073	313.2 (294.8–331.6)	<0.001	178.7 (161.0–196.4)	<0.001
Elevated-increasing group	696	489.3 (462.5–516.1)	<0.001	297.7 (272.2–323.2)	<0.001
Elevated-decreasing group	1534	441.0 (418.7–463.3)	<0.001	263.5 (241.8–285.2)	<0.001
Progression of AS (*n* = 10,208)					
Low-stable group	441	Ref.		Ref.	
Moderate-stable group	3439	24.9 (12.9–36.8)	<0.001	19.2 (7.0–31.4)	<0.001
Elevated-stable group	5028	48.7 (36.7–60.7)	<0.001	36.4 (23.8–49.0)	<0.001
Elevated-increasing group	363	69.7 (52.4–86.9)	<0.001	51.2 (33.2–69.1)	<0.001
Elevated-decreasing group	937	67.0 (52.8–81.2)	<0.001	48.4 (33.3–63.5)	<0.001

Abbreviations: β, regression coefficient; baPWV, brachial–ankle pulse wave velocity; SBP, systolic blood pressure. Model 1 for the baseline baPWV was unadjusted. Model 2 for the baseline baPWV was adjusted for age, gender, education level, smoking status, alcohol drinking status, physical activity, BMI, MAP, FBG, and TC. Model 1 for the progressive baPWV was adjusted for the baseline baPWV. Model 2 for the progressive baPWV was adjusted for age, gender, education level, smoking status, alcohol drinking status, physical activity, baseline baPWV, BMI, MAP, FBG, and TC.

## Data Availability

The data supporting the reported results are available on request from the corresponding author (Y.H.-T.).

## Supplementary Materials

The following supporting information can be downloaded at: https://www.mdpi.com/article/10.3390/ijerph191610046/s1.
