# Supplementary material for: Systolic Blood Pressure Trajectories and the Progression of Arterial Stiffness in Chinese Adults
Source: Int J Environ Res Public Health. 2022 Aug 15;19(16):10046. doi: 10.3390/ijerph191610046 (PMC9408732; doi:10.3390/ijerph191610046)
Supplement: Supplementary file 1 [file ijerph-19-10046-s001.zip › ijerph-1857890-supplementary.pdf]

Association of Blood Pressure Trajectories with Arterial Stiffness Progression

Short title: Blood Pressure Trajectories and arterial stiffness

Supplementary Table S1. Characteristics of participants with and without repeated baPWV measurements.

Variables	With repeated baPWV measurements	Without repeated baPWV measurements
No. of participants	14,223	16,161
Age, years	47.8 ± 11.4	49.0 ± 11.7
Gender, n (%)		
Male	8,787 (61.8)	10,020 (62.0)
Female	5,436 (38.2)	6,141 (38.0)
Education, n (%)		
Low	521 (3.7)	597 (3.7)
Intermediate	7,916 (55.6)	8,986 (55.6)
High	5,786 (40.7)	6,578 (40.7)
Physical activity, n (%)		
Inactive	12,641 (88.9)	14,351 (88.8)
Active	1,582 (11.1)	1,810 (11.2)
Smoking, n (%)		
Never	9,020 (63.4)	1,0262 (63.5)
Former	779 (5.5)	873 (5.4)
Current	4,424 (31.1)	5,026 (31.1)
Drinking, n (%)		
Never	7,750 (54.5)	8,792 (54.4)
Former	468 (3.4)	549 (3.4)
Current	6,005 (42.2)	6,820 (42.2)
BMI, kg/m^2	24.8 ± 3.4	25.0 ± 3.4
MAP, mmHg	101.6 ± 10.8	103.4 ± 11.3
FBG, mmol/L	5.3 ± 1.2	5.4 ± 1.3
TC, mmol/L	4.9 ± 1.0	4.9 ± 1.0

Abbreviations: BMI, body mass index; MAP, mean arterial pressure; FBG: fasting blood glucose; SBP: systolic blood pressure; TC, total cholesterol.

Continues variables displayed as means ± standard deviation (SD), and categorical variables are displayed as numbers (percentages).

Supplementary Table S2. Associations of SBP trajectories with baseline baPWV stratified by age.

Trajectory patterns	No. of participants	Model 1		Model 2	
		β (95% CI)	P-value	β (95% CI)	P-value
Age < 50 years					
Low-stable group	4,016	Ref.		Ref.	
Moderate-stable group	9,162	177.0 (168.0-186.0)	<0.001	81.1 (70.3-91.9)	<0.001
Elevated-stable group	3,413	361.0 (349.9-372.1)	<0.001	188.9 (171.8-206.0)	<0.001
Elevated-increasing group	438	487.3 (454.4-502.3)	<0.001	304.0 (278.2-329.8)	<0.001
Elevated-decreasing group	605	523.8 (503.0-544.5)	<0.001	289.3 (259.4-319.1)	<0.001
Age ≥ 50 years					
Low-stable group	2,921	Ref.		Ref.	
Moderate-stable group	5,900	199.1 (183.4-214.7)	<0.001	139.8 (122.3-157.3)	<0.001
Moderate-increasing group	1,294	438.5 (415.4-461.6)	<0.001	318.5 (291.8-345.2)	<0.001
Elevated-decreasing group	1,957	363.2 (343.0-383.4)	<0.001	218.7 (187.5-249.9)	<0.001
Elevated-stable group	678	541.7 (512.2-571.2)	<0.001	348.5 (306.2-390.7)	<0.001

Abbreviations: β, indicates regression coefficient; baPWV, brachial-ankle pulse wave velocity; SBP: systolic blood pressure.

Model 1 was unadjusted.

Model 2 was adjusted for age, gender, education level, smoking status, alcohol drinking status, physical activity, BMI, MAP, FBG, and TC.

Supplementary Table S3. Associations of SBP trajectories with baPWV progression stratified by age.

Trajectory patterns	No. of participants	Model 1		Model 2	
		β (95% CI)	P-value	β (95% CI)	P-value
Age < 50 years					
Low-stable group	1,766	Ref.		Ref.	
Moderate-stable group	4,457	33.6 (26.5-40.8)	<0.001	24.4 (16.7-32.1)	<0.001
Moderate-increasing group	2,321	62.9 (54.2-71.6)	<0.001	44.1 (34.2-54.1)	<0.001
Elevated-stable group	607	97.0 (84.1-109.9)	<0.001	74.0 (46.1-101.9)	<0.001
Elevated-decreasing group	96	90.0 (63.3-116.7)	<0.001	81.0 (66.8-95.1)	<0.001
Age ≥ 50 years					
Low-stable group	983	Ref.		Ref.	
Moderate-stable group	2,354	22.8 (12.3-33.4)	<0.001	22.7 (11.8-33.7)	<0.001
Moderate-increasing group	537	46.1 (26.2-65.9)	<0.001	46.2 (25.7-66.8)	<0.001
Elevated-decreasing group	820	43.5 (27.8-59.1)	<0.001	42.6 (26.3-58.8)	<0.001
Elevated-stable group	282	63.4 (39.7-67.1)	<0.001	50.5 (36.1-64.9)	<0.001

Abbreviations: β, indicates regression coefficient; baPWV, brachial-ankle pulse wave velocity; SBP: systolic blood pressure.

Model 1 was adjusted for baseline baPWV.

Model 2 was adjusted for age, gender, education level, smoking status, alcohol drinking status, physical activity, baseline baPWV, BMI, MAP, FBG, and TC.

Supplementary Table S4. Associations of SBP trajectories with baseline baPWV stratified by gender.

Trajectory patterns	No. of participants	Model 1		Model 2	
		β (95% CI)	P-value	β (95% CI)	P-value
Male					
Low-stable group	6,945	Ref.		Ref.	
Moderate-stable group	9,523	179.2 (169.3-189.1)	<0.001	111.1 (99.7-122.5)	<0.001
Elevated-decreasing group	2,437	361.5 (346.7-376.2)	<0.001	195.6 (173.7-217.5)	<0.001
Moderate-increasing group	1,529	433.2 (415.5-450.9)	<0.001	273.0 (253.6-292.4)	<0.001
Elevated-stable group	766	539.6 (515.8-563.4)	<0.001	301.1 (269.4-332.7)	<0.001
Female					
Low-stable group	3,370	Ref.		Ref.	
Moderate-stable group	3,779	211.1 (198.0-224.2)	<0.001	88.8 (74.6-103.0)	<0.001
Moderate-increasing group	1,157	500.3 (481.5-519.2)	<0.001	229.0 (205.8-252.3)	<0.001
Elevated-decreasing group	488	569.1 (542.3-595.9)	<0.001	211.2 (174.8-247.6)	<0.001
Elevated-stable group	390	766.9 (737.3-796.5)	<0.001	398.5 (359.8-437.2)	<0.001

Abbreviations: β, indicates regression coefficient; baPWV, brachial-ankle pulse wave velocity; SBP: systolic blood pressure.

Model 1 was unadjusted.

Model 2 was adjusted for age, gender, education level, smoking status, alcohol drinking status, physical activity, BMI, MAP, FBG, and TC.

Supplementary Table S5. Associations of SBP trajectories with baPWV progression stratified by gender.

Trajectory patterns	No. of participants	Model 1		Model 2	
		β (95% CI)	P-value	β (95% CI)	P-value
Male					
Low-stable group	2,720	Ref.		Ref.	
Moderate-stable group	4,087	21.6 (14.5-28.7)	<0.001	18.0 (10.6-25.3)	<0.001
Elevated-decreasing group	1,036	52.5 (39.4-65.7)	<0.001	44.2 (30.6-57.7)	<0.001
Moderate-increasing group	621	64.5 (47.2-81.9)	<0.001	54.7 (43.4-66.0)	<0.001
Elevated-stable group	323	65.3 (54.5-76.0)	<0.001	55.6 (37.7-73.5)	<0.001
Female					
Low-stable group	1,981	Ref.		Ref.	
Moderate-stable group	2,327	29.2 (22.2-36.1)	<0.001	23.7 (16.5-30.9)	<0.001
Moderate-increasing group	697	58.3 (47.3-69.4)	<0.001	41.4 (29.6-53.2)	<0.001
Elevated-decreasing group	220	75.8 (58.8-92.8)	<0.001	51.6 (33.6-69.6)	<0.001
Elevated-stable group	211	62.9 (44.5-81.2)	<0.001	56.0 (36.9-75.0)	<0.001

Abbreviations: β, indicates regression coefficient; baPWV, brachial-ankle pulse wave velocity; SBP: systolic blood pressure.

Model 1 was adjusted for baseline baPWV.

Model 2 was adjusted for age, gender, education level, smoking status, alcohol drinking status, physical activity, baseline baPWV, BMI, MAP, FBG, and TC.

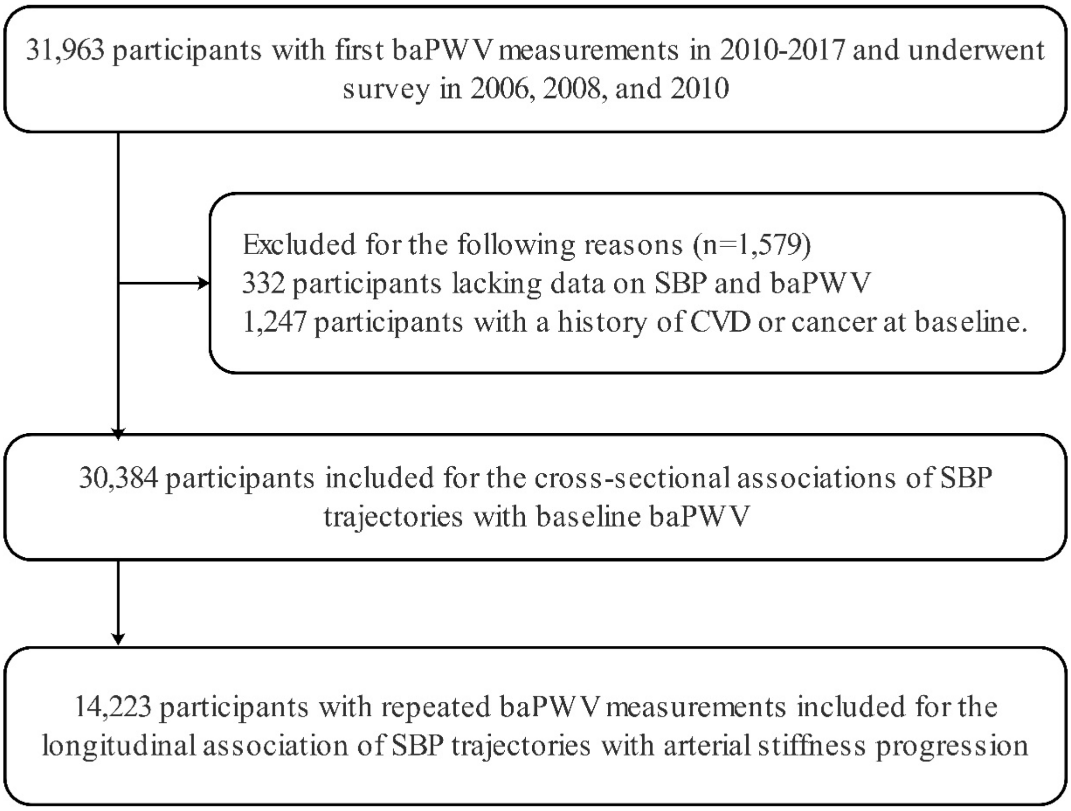

Supplementary Figure S1. Flowchart of the study population

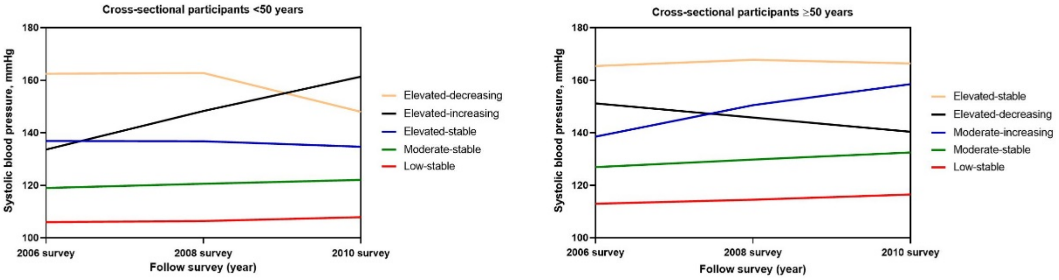

Supplementary Figure S2. SBP trajectory patterns for 30,384 participants with baseline baPWV during 2006–2010 stratified by age. SBP was classified into five groups according to the latent mixture modeling. The x-axis refers to the years of follow survey of the participants. The y-axis refers to the SBP concentrations of the participants.

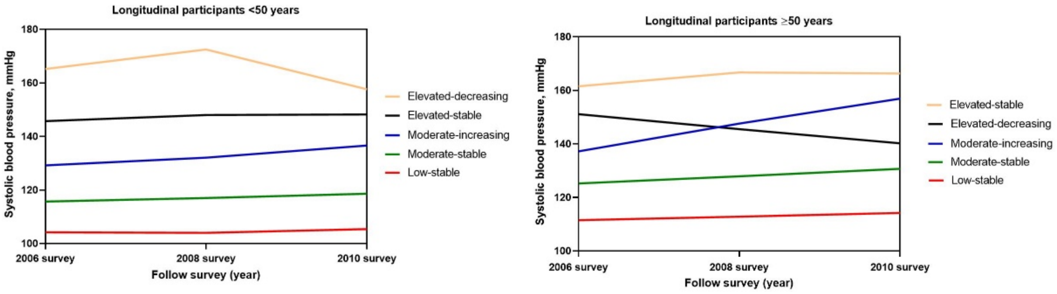

Supplementary Figure S3. SBP trajectory patterns for 14,223 participants with repeated baPWV during 2006–2010 stratified by age. SBP was classified into five groups according to the latent mixture modeling. The x-axis refers to the years of follow survey of the participants. The y-axis refers to the SBP concentrations of the participants.

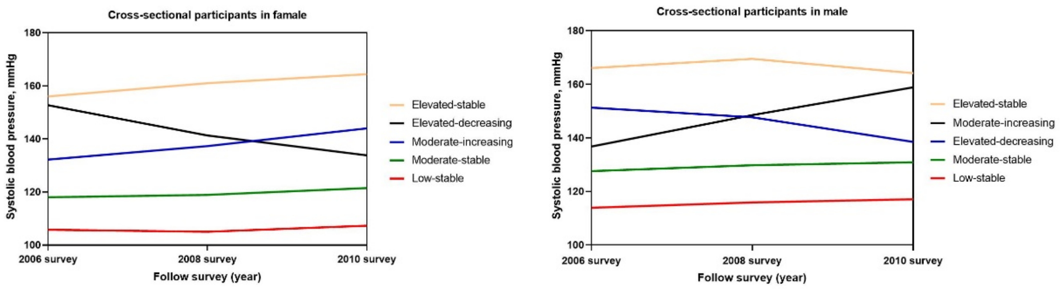

Supplementary Figure S4. SBP trajectory patterns for 30,384 participants with baseline baPWV during 2006–2010 stratified by gender. SBP was classified into five groups according to the latent mixture modeling. The x-axis refers to the years of follow survey of the participants. The y-axis refers to the SBP concentrations of the participants.

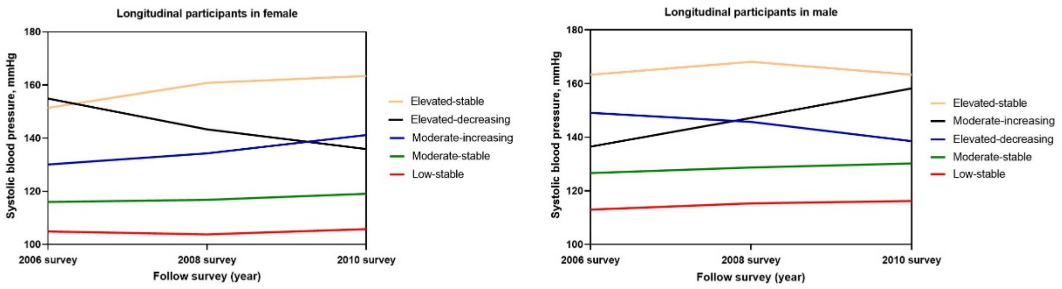

Supplementary Figure S5. SBP trajectory patterns for 14,223 participants with repeated baPWV during 2006–2010 stratified by gender. SBP was classified into five groups according to the latent mixture modeling. The x-axis refers to the years of follow survey of the participants. The y-axis refers to the SBP concentrations of the participants.
